# Medical Opinion and Movement

**Published:** 1911-06-10

**Authors:** 


### MEDICAL OPINION AND MOVEMENT.

### Gonorrhœal Iritis.

THE use of gonococcal vaccines for the various manifestations of gonorrhœa has been the subject of widely divergent opinions. It seems now to be generally thought that for the acute urethritis they are useless, if not worse; but that for some of the later and chronic lesions caused by the diplococcus of Neisser vaccine treatment succeeds where no other kind will. In the *Archives of the Middlesex Hospital* Mr. Lang expresses his high opinion of massive doses of this vaccine for all cases of iritis which can be traced to a gonorrhœal origin. They cut short the pain and duration of the iritis; rheumatic pains and swollen joints are also dispersed at the same time. Up to the present he has not had a case of relapse, but as the first cases were treated only three years ago, he feels it a little early to say with confidence that absolute cure is always attained. To ensure the best results Mr. Lang recommends massive and increasing doses. Two doses of two hundred, of three hundred, and of five hundred millions are given at intervals of a week; and a final dose of one thousand millions, and even a further one half as large again, may end the course of treatment. These enormous doses are given only when the eye is alone affected; when there are other lesions elsewhere smaller quantities are injected. There should be no reaction, either at the seat of injection or constitutionally as a result of it.

### External Application of Cold.

In the *Münchener Medizinische Wochenschrift* Dr. Riehl contributes an account of his researches to determine the effects of cold external applications in lowering the internal temperature of the body, especially in regard to the stomach. For this purpose a maximal thermometer was used, measuring 9½ cm. long and 0.5 cm. thick, and capable of measuring a range of temperature from 32° to 40° C. This thermometer was fixed into the eye of a gastric sound so that the lower end projected outside the tube. The experiments were made with individuals already accustomed to have the sound passed, so that it could be effected rapidly and the results not vitiated by too long a sojourn in the œsophagus. The sound was passed rapidly into the stomach, left there for ten minutes, and then rapidly withdrawn. The rectal temperature was taken at the same time. The temperature was in this way taken several times, both before and during the application of cold externally. The lowest temperature recorded was 35°.7 C. in the stomach and 36°.7 C. in the rectum, showing a lowering of temperature of 1°.8 C. for the stomach and 1°.1 C. for the rectum by cold external application. These experiments confirm the efficacy of the application of ice to the abdomen in cases of gastric hæmorrhage, as such a lowering of internal temperature must tend to promote blood coagulation both by the ischæmia produced and by the reflex action on the vaso-constrictor nerves.

### The Diplobacillus of Conjunctivitis.

IN the *Klinische Monatsblätter für Augenheilkunde* there is an interesting report on some research work carried out by Dr. Reis at the ophthalmic clinic of the University of Lemberg on the pathology of the Morax-Axenfeld diplobacillus and its relation to conjunctivitis. Of three hundred cases of catarrhal, phlyctenular, or granular conjunctivitis treated in the course of six months, eighty-five showed the presence of this diplobacillus as the pathogenic agent. Seventy of them were of the chronic or subacute catarrhal type among a total of 163—that is, nearly 50 per cent. The chief clinical sign by which this form of conjunctivitis may be recognised consists in its preference for the internal angle of the eye as a starting-point of infection. In this way a diagnosis was made clinically in about two-thirds of the cases, afterwards confirmed by bacteriological examination. On the other hand, the diplobacillus was found in twenty cases in which it was not suspected clinically, and in ten cases it was not found present when it was suspected. The correct diagnosis is of importance, as sulphate of zinc, which is of small value against other forms of conjunctivitis, is almost specific against the diplobacillus. According to the author the diagnosis is particularly difficult and the signs misleading in cases of mixed infections. The author finds that the blood or conjunctival secretion produces no bacteriolytic effect upon the diplobacillus, so that medicaments are not effective by reason of the hyperæmia they may produce, and consequent passage of bacteriolytic or opsonic bodies into the conjunctival sac. On the other hand, the serum of immunised rabbits shows highly developed bacteriolytic and agglutinating properties. The author suggests therefore that the only rational specific treatment would be the instillation of immunised serum, so as to bring it into direct contact with the pathogenic germs.

### Atropine and Dysmenorrhœa.

DR. DRENKHAHN writes enthusiastically of the value of atropine to control uterine contractions in conditions of dysmenorrhœa and also in conditions of puerperal infection, in which it is desirable to subdue uterine contractions and keep the organ at rest. The value of belladonna or atropine for the control of painful spasm in unstriped muscle has long been familiar in other regions of the body. The drug may be administered locally by injecting an aqueous solution of atropine (0 gr. 0.001 milligr. atropine in 1 c.c. aqua) into the cervical canal, or tampons of a 1 per cent. solution may be introduced into the vagina and lodged into the posterior vaginal cul-de-sac. Dr. Novak, of Vienna, on the other hand, obtains equally good results in cases of dysmenorrhœa by avoiding any local interference and administering the drug in the form of pilules by the mouth. He reports five cases in which successful results attended this mode of treatment

